# Metropolitan and Town Sewerage
*Metropolitan and Town Sewage; their Value and Disposal. By A. Sayer, M.D. London: Calder. 1857.


**Published:** 1857-07

**Authors:** 


### METROPOLITAN AND TOWN SEWERAGE.*

Few men have paid so much earnest attention to the sewerage question as Dr. Sayer. In his present work he supplies some most valuable and curious information. The following passages give the pith of Dr. Sayer's practical views:—

"No human foresight can possibly assign limits to the growing extent and population of London,—therefore any *scheme*, that provides only for a limited prospective period of time, is unworthy of the present era, and not commensurate with the future.—No

---

* Metropolitan and Town Sewage; their Value and Disposal. By A. Sayer, M.D. London: Calder. 1857.

*scheme* that does not contemplate all present and possible future localities of London, that cannot minister to all levels and falls east and west of the whole water-shed areas, north and south of the Thames, is not commensurate with the requirements, present or prospective, of her population.

"Is not the time at hand, when adequate steam power should transfer to numerous covered reservoirs and covered filtering beds, within our agricultural districts, near and remote, through closed cast-iron channels, receiving in their course daily abundant tributary streams from the metropolis and all large towns, '*sewage waters*,' to spread by irrigation, fertility and minister to increasing abundance?

"Our public roads,—the net-work of modern railways, will indicate the appropriate lines,—and the electric telegraph will minister its ubiquity, to govern and regulate valves for transmissive circulation. Should not the metropolis initiate a great system, in union and co-operation with our sister towns, to '*restore*' to the land that which modern domestic adaptations have taken from our soil, in *violation* of the *beneficent* law imposed on all human and animal existence, which ordained,—that by the multiplication of living creatures, '*the earth is to be replenished*' with the elements of ever renewed and increasing fertility?

"Poor and rich, the peasant and the peer, all are equally interested in the great undertaking, pregnant as it is with more abundant crops, more numerous herds, plenty and price, content and happiness."

